# Atlantoaxial rotatory fixation as a rare complication from head positioning in otologic surgery: Report of two cases in young children

**DOI:** 10.1186/s13037-016-0116-7

**Published:** 2017-02-01

**Authors:** Hiroshi Sakaida, Koji Akeda, Akihiro Sudo, Kazuhiko Takeuchi

**Affiliations:** 1 Department of Otorhinolaryngology – Head & Neck Surgery, Mie University Graduate School of Medicine, 2-174 Edobashi, Tsu, Mie 514-8507 Japan; 2 Department of Orthopaedic surgery, Mie University Graduate School of Medicine, Tsu, Mie Japan

**Keywords:** Adverse event, Atlantoaxial rotatory fixation, Otologic surgery, Patient safety, Torticollis

## Abstract

**Background:**

Atlantoaxial rotatory fixation is a condition in which the first and second vertebrae of the cervical spine become interlocked in a rotated position. This condition can result in serious consequences and thus have a significant impact on patients, especially when diagnosis and treatment are delayed. Some cases of atlantoaxial rotatory fixation have been described in association with otologic surgery or plastic surgery involving the ear. We present the cases of two pediatric patients who developed atlantoaxial rotatory fixation following otologic surgery and we review the relevant literature.

**Case presentation:**

One patient was a 7-year-old boy who underwent tympanoplasty for cholesteatoma. The other patient was a 5-year-old girl with profound sensorineural hearing loss who underwent cochlear implantation. Both patients developed atlantoaxial rotatory fixation on the day after surgery, and they were treated conservatively. Our literature search using relevant terms identified 12 similar published cases. Thus, a total of 14 patients, including our 2 patients, were evaluated. Most of the patients were children and typically they complained of painful torticollis and exhibited a characteristic posture called the “cock-robin” position on the day after surgery. Mostly, the direction of torticollis was opposite to the side of surgery. Most of the patients received conservative treatment alone, but three underwent surgical treatment.

**Conclusion:**

The correlation between the direction of torticollis and the side of surgery suggests that rotation of the head during surgery has an impact on development of postoperative atlantoaxial rotatory fixation. Thus, children undergoing otologic surgery are thought to be at a risk of postoperative atlantoaxial rotatory fixation. Although rare, the surgical team needs to be aware of this adverse event and pay close attention to this possibility throughout the perioperative period. Perioperative management should include informed consent, preoperative assessment of the range of head and neck motion, proper intraoperative positioning and monitoring of the position, and postoperative follow-up. Postoperative atlantoaxial rotatory fixation is not completely preventable, but good perioperative management can minimize the damage resulting from this condition.

**Electronic supplementary material:**

The online version of this article (doi:10.1186/s13037-016-0116-7) contains supplementary material, which is available to authorized users.

## Background

Patient positioning during surgery is determined by balancing adequate exposure and access to the target area with the risks related to the position. Inappropriate positioning can lead to complications. In otologic or otoplastic surgery, the head is typically rotated toward the opposite side to obtain optimal access to the temporal bone region. Although rare, this position has been associated with atlantoaxial rotationally fixation (AARF) or atlantoaxial rotationally subluxation (AARS) [1–10], in which the anterior facet of the first cervical vertebrae (C1) becomes locked on the facet of the second cervical vertebrae (C2), and rotation of the C1-C2 joint becomes limited.

AARF can result in serious consequences and thus have a significant impact on patients, especially when diagnosis and treatment are delayed [6, 11, 12]. Cases of AARF following otologic surgery have been sporadically reported, but the clinical features remain unclear. Here, we present the cases of two children who developed AARF following otologic surgery, and we review published data for this condition. Perioperative patient management for avoidance of AARF is also discussed.

## Case presentation

### Case 1

**Fig. 1:**
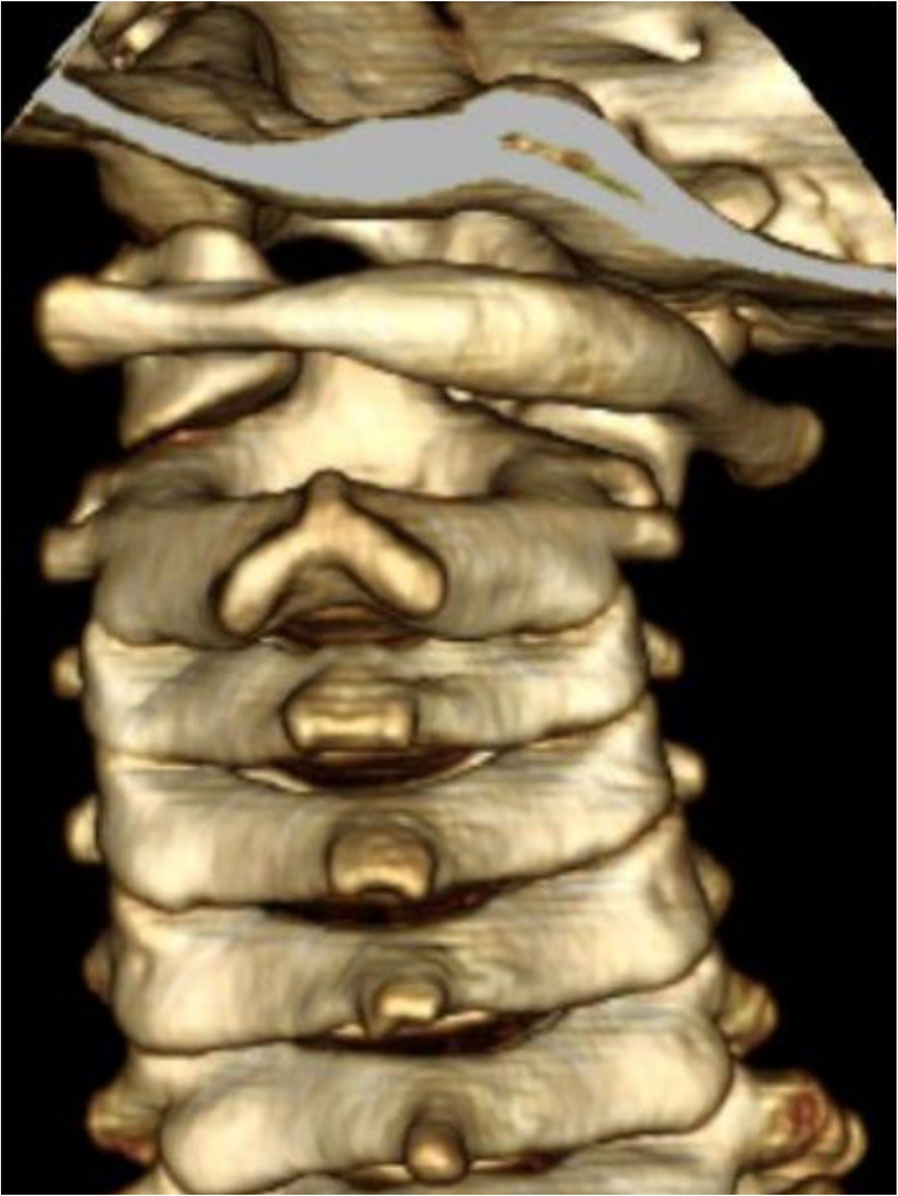
A posterior view of three-dimensional reconstruction computed tomography of the cervical spine in case 1, demonstrating the interlocked atlantoaxial joint in a rotatory position to the left

A 7-year-old boy (115 cm, 21 kg) was referred to our department for treatment of middle ear cholesteatoma in his right ear. Tympanoplasty was performed under general anesthesia with the neck rotated toward the left side. The surgery took 3 h 34 min and was completed uneventfully. There were no abnormalities during and on awakening from general anesthesia. The following day, the patient complained of severe neck pain without obvious neurological abnormalities. His neck was fixed in a position rotated to the left and tilted slightly to the right. We asked for an opinion from an orthopedic surgeon, who pointed out AARF on the basis of the characteristic posture. Computed tomography (CT) of the cervical spine revealed that the C1-C2 joint was locked, with C1 rotated toward the left and tilted to the right (Fig. 1). This observation established a diagnosis of AARF. The patient was treated conservatively with traction and application of a soft cervical collar. His condition improved with 5 days of treatment and he was discharged to home 7 days after surgery.

Two years later, the patient, who was then 9 years old (125 cm, 25 kg), again underwent tympanoplasty for recurrent cholesteatoma in his right ear. At this time, we carefully ensured that the rotation angle of the head was kept within 45° and we used sandbags to prevent further rotation. The surgery took 2 h 50 min. On awaking from general anesthesia, the patient had no sign of AARF. On the following day, the patient complained of neck pain without neurological symptoms, and his neck was again rotated to the left and tilted slightly to the right. A diagnosis of AARF was established based on physical examination and CT scans. The patient received the same treatment as previously performed, after which he became symptom-free and was discharged 1 week after surgery.

### Case 2

The second patient was a 5-year-old girl (113 cm, 19 kg) with bilateral profound sensorineural hearing loss. She had no underlying abnormalities except for hearing loss. Cochlear implantation in the left ear took 3 h 46 min and was successfully performed. The patient awoke from general anesthesia without any sign of AARF. The following day, she complained of neck pain and a diminished range of motion. Her head position was tilted to the left and rotated to the right. No neurological abnormalities were present. The patient was examined by an orthopedic surgeon and underwent CT to check the C1-C2 joint, which confirmed AARF. After conservative treatment with application of a soft cervical collar, she was discharged to home 1 week after surgery with full resolution of symptoms and pain.

### Safety precautions

After the first episode of AARF in case 1, we introduced safety precautions to prevent AARF in pediatric patients undergoing any type of otologic surgery. After induction of general anesthesia, the head is rotated toward the opposite side. The angle of rotation is limited to within 45°, with sandbags of 1 kg placed beside the head to prevent further rotation.

### Review of the literature

A PubMed search limited to articles in English was undertaken using the terms “atlantoaxial rotationally fixation” (AARF), “atlantoaxial rotationally subluxation” (AARS), “Grisel’s syndrome”, “otologic surgery”, “tympanoplasty”, “tympanomastoidectomy”, “cochlear implantation”, and “otoplasty”, for publication dates from January 1990 to April 2016. Several names, including AARF, AARS, and Grisel’s syndrome, have been used for a disorder with impaired motion of the C1-C2 joint. Therefore, these were included in a single category. The search generated case reports, case series and reviews, which were assessed for content and relevance. Among articles describing cases of AARF, AARS, or Grisel’s syndrome, those associated with otologic or plastic surgery involving the ear were selected. Cases without detailed information on age, sex, underlying disease, and type of surgery were excluded.

Our search of the literature identified 12 cases [1–10]. With inclusion of our two patients, the clinical characteristics of a total of 14 patients were reviewed [see Additional file 1]. Age ranged from 5 to 28 years, and all patients except one were children. Twelve of the 14 patients had no underlying predisposing conditions for AARF. One patient had trisomy 21 and another had a history of craniofacial surgery for sagittal craniosynostosis. The conditions for which surgery was performed included otitis media, cholesteatoma, profound sensorineural hearing loss, and cryptotia. Therefore, several types of surgery were performed. All patients presented with a typical “cock-robin” posture. The timing of onset of AARF was not clearly documented in one patient. In the other 13 patients, AARF developed immediately after surgery in 2 cases, on the day after surgery in 10 cases, and 3 days after surgery in one case.

The duration of symptoms before diagnosis varied from a few hours to 6 months. The side of surgery or the direction of torticollis was not clearly documented in 4 patients. In nine of the other 10 patients, the direction of torticollis was opposite to the side of surgery. Twelve cases were diagnosed using physical examination and CT scans and 2 were diagnosed on plain radiographs. All patients initially received conservative treatment with traction and application of a soft collar. Two patients experienced recurrence after a second surgery, even with taking of precautions. Three patients ultimately underwent surgical fixation of the C1-C2 joint because of instability of the joint and chronically repeated AARF.

## Discussion

The C1-C2 joint is responsible for up to 60% of total rotation in the craniocervical region [13]. C1 and C2 form a pivotal joint that is unique with regard to its bony anatomy, joint shape, and orientation [14]. C1 is a ring-shaped structure that articulates superiorly with the occipital condyles and inferiorly with C2 via the atlantoaxial joint [14]. C2 has the odontoid process that arises perpendicularly from the upper aspect of the body. Several ligaments connect C1, C2, and the occipital bone in a complex manner [14]. These ligaments play important anatomical and biomechanical roles that stabilize the joint while limiting excessive motion [14].

AARF can occur spontaneously or as a result of trauma to the head and neck region, including falls, low-speed vehicular accidents, and contact sports [12, 15]. AARF also occurs in association with upper respiratory infection or otolaryngological surgery [2, 12, 16], and is more common in children than adults, regardless of the cause [12]. Historically, AARF has been classified into four types according to the presence, direction, and degree of displacement between C1 and C2 [17]. A new classification system based on motion analysis using CT imaging was proposed by Pang et al. [18]. The prognosis of AARF differs depending on the type [12], and thus determining the type is important from a therapeutic perspective. However, patients with all types of AARF initially present with a characteristic painful “cock-robin” posture, in which the head is rotated in one direction and tilted in the opposite direction.

Otologic surgery has features that can induce AARF. Unlike neurosurgical procedures, the head is not rigidly fixed with pins; instead, it is simply placed on a positioning pad. Due to decreased muscle tone and loss of consciousness during general anesthesia, the surgical procedure or placement of the surgeon’s hands on the head may produce a force that causes excessive passive motion of the head. As the head excessively rotates, the weight of the head itself may facilitate further rotation.

Grisel’s syndrome has been used to refer collectively to AARF associated with upper respiratory infection and otolaryngological surgery [2, 16, 19, 20]. The most common surgical cause of Grisel’s syndrome is tonsillectomy/adenotonsillectomy, whereas otologic surgery is a less common cause [2, 16]. Both procedures are classified as otolaryngological surgery, but they differ in patient positioning during surgery and direct application to the pharynx. Several pathogeneses for Grisel’s syndrome have been proposed [21], but the exact pathogenesis is unclear. Currently, the most widely believed pathogenesis is hematogenous spread of infection from the posterior-superior pharynx to the cervical spine [19, 21]. The resultant increased flexibility of ligaments around the C1-C2 joint is thought to cause subluxation and subsequent fixation of the joint. This hypothesis is plausible and explains the pathogenesis of AARF after upper respiratory infection or tonsillectomy/adenotonsillectomy, in which surgery is directly applied to the pharynx. However, the pathogenesis of AARF following otologic surgery does not fit this hypothesis, because surgery is not performed directly on the pharynx. It is also difficult to explain how middle ear inflammation spreads directly to the pharyngovertebral plexus. In addition, AARF can occur after non-otolaryngological procedures [22–24], which suggests that inflammatory spread to the pharyngovertebral plexus is not essential for development of postoperative AARF.

Our literature review showed that the direction of torticollis is strongly correlated with the side of surgery in cases of AARF following otologic surgery. This directional correlation indicates that certain mechanical stress on the joint influences development of postoperative AARF. Pang et al. suggested that torticollis associated with nasopharyngeal infection was likely to be muscular reflex, rather than true AARF [12], and described head positioning during surgery and general anesthesia as contributory factors to postoperative AARF. We also believe that AARF following otologic surgery results from excessive rotation of the head under general anesthesia, in which the protective function of the musculature is lacking. In addition, because two patients in our review repeatedly developed AARF, individual vulnerability to rotatory movement of the head may also contribute to development of AARF.

Issues regarding patient positioning during surgery are mainly described in the nursing literature [25]. However, otologic surgery-associated AARF has rarely been described, probably because it is a rare occurrence or it is merely underreported. Children undergoing otologic surgery under general anesthesia are at higher risk of AARF. In 2010, Kim et al. proposed the first guidelines for perioperative care of patients with a risk of AARF [8]. We are basically in agreement with the proposal [8], but we would like to add some practical measures to prevent AARF from the perspective of otologic surgeons. First, informed consent should include the possibility of postoperative AARF, although it is a rare occurrence. This is because AARF can have a significant impact on the patient due to prolonged hospitalization, a need for treatment, and the possibility of disastrous sequelae. Preoperatively, the positon and range of motion of the head and neck should be assessed. In the operating room, patient positioning should be determined by balancing surgical access with possible complications resulting from the position. Kim et al. recommended that rotation of the neck should be less than 60° [8]. Individual difference of patients should be taken into consideration and positioning needs to be checked by other members of the surgical team. If more rotation is needed, a roll or pillow placed under the shoulder on the side of rotation is recommended. Tilting the operating table and adjusting its height are other options to obtain optimum access to target sites.

Once appropriate positioning is determined before surgery, preventative measures to limit further rotation should be considered. In our hospital, sandbags of 1 kg are placed beside the head to prevent further rotation. During surgery, care should be taken not to apply excessive force to the head in the surgical procedure or with the surgeon’s hands. The head position must be maintained throughout surgery by regular monitoring. Postoperatively, the position and range of movement of the head and neck should be checked repeatedly at least until the day after surgery because AARF often develops on this day. If the patient complains of neck pain or there is a sign suggestive of AARF, prompt orthopedic or neurosurgical consultation is strongly recommended because a delay in diagnosis may worsen the prognosis and have harmful consequences [11, 12]. CT is essential for diagnosis of AARF, and Pang et al. recommended a 3-position CT protocol to study C1-C2 rotatory dynamics and grade the severity of AARF [12, 18]. The World Health Organization has advocated safe surgery and issued guidelines to promote such surgery [26]. The guidelines do not explicitly describe adverse events associated with patient positioning, but the surgical team should ensure patient safety through effective communication and exchange of critical information.

AARF can be managed with conservative treatment and surgical intervention, and traction is the initial treatment of choice [12]. Our review showed that most patients recovered well with conservative treatment alone. Surgery is usually reserved for cases with recurrent dislocation and incomplete improvement [8, 12, 27].

Some limitations must be considered when interpreting the present results. Because our review of the literature spanned approximately 25 years, the clinical background, diagnostic criteria, and therapeutic approach may have differed considerably among cases. Due to this heterogeneity, accurate comparison between cases is difficult. In addition, this kind of adverse event may be underreported, probably because it is not directly associated with outcomes of the surgery itself, or there may be other barriers that prevent reporting of adverse events. Due to potential reporting bias, the incidence of AARF following otologic surgery is unknown. Nonetheless, this condition is a possibility, and otologists and other members of the surgical team need to be aware of AARF as a rare but potentially disastrous complication.

## Conclusion

We have described the cases of two children who developed AARF following otologic surgery. A review of similar published cases suggested that AARF following otologic surgery occurs mainly in children, and that symptoms develop mostly on the day after surgery. Both head positioning during surgery and individual vulnerability may contribute to development of AARF. Perioperative patient safety requires a team of surgeons, anesthesiologists, nurses, and other healthcare personnel to prevent AARF and minimize damage if this condition occurs.

## Additional file

Additional file 1: Data for 14 patients. Abbreviations: M, male; F, female; ND, not documented; SNHL, sensorineural hearing loss; POD, postoperative day. (PPTX 52 kb)

## Abbreviations

AARF: Atlantoaxial rotationally fixation
AARS: Atlantoaxial rotationally subluxation
