# Supplementary material for: Atlantoaxial rotatory fixation as a rare complication from head positioning in otologic surgery: Report of two cases in young children
Source: Patient Saf Surg. 2017 Feb 1;11:5. doi: 10.1186/s13037-016-0116-7 (PMC5289022; doi:10.1186/s13037-016-0116-7)
Supplement: Additional file 1: — Data for 14 patients. Abbreviations: M, male; F, female; ND, not documented; SNHL, sensorineural hearing loss; POD, postoperative day. (PPTX 52 kb) [file 13037_2016_116_MOESM1_ESM.pptx]

## Slide 1
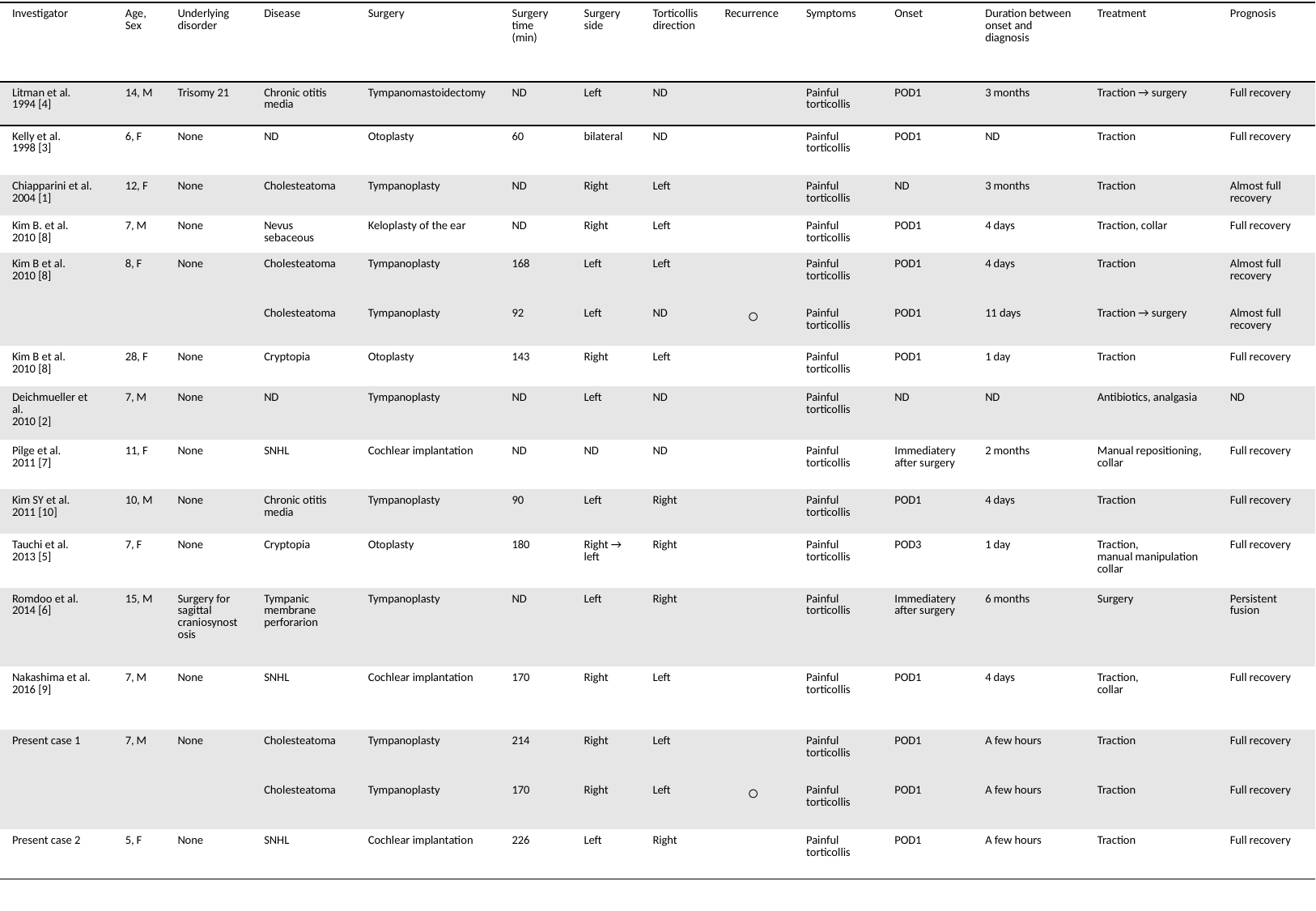

| Investigator | Age, Sex | Underlying disorder | Disease | Surgery | Surgery time (min) | Surgery side | Torticollisdirection | Recurrence | Symptoms | Onset | Duration between onset and diagnosis | Treatment | Prognosis |
| --- | --- | --- | --- | --- | --- | --- | --- | --- | --- | --- | --- | --- | --- |
| Litman et al. 1994 [4] | 14, M | Trisomy 21 | Chronic otitis media | Tympanomastoidectomy | ND | Left | ND | | Painful torticollis | POD1 | 3 months | Traction → surgery | Full recovery |
| Kelly et al. 1998 [3] | 6, F | None | ND | Otoplasty | 60 | bilateral | ND | | Painful torticollis | POD1 | ND | Traction | Full recovery |
| Chiapparini et al. 2004 [1] | 12, F | None | Cholesteatoma | Tympanoplasty | ND | Right | Left | | Painful torticollis | ND | 3 months | Traction | Almost full recovery |
| Kim B. et al. 2010 [8] | 7, M | None | Nevus sebaceous | Keloplasty of the ear | ND | Right | Left | | Painful torticollis | POD1 | 4 days | Traction, collar | Full recovery |
| Kim B et al. 2010 [8] | 8, F | None | Cholesteatoma | Tympanoplasty | 168 | Left | Left | | Painful torticollis | POD1 | 4 days | Traction | Almost full recovery |
| | | | Cholesteatoma | Tympanoplasty | 92 | Left | ND | 〇 | Painful torticollis | POD1 | 11 days | Traction → surgery | Almost full recovery |
| Kim B et al. 2010 [8] | 28, F | None | Cryptopia | Otoplasty | 143 | Right | Left | | Painful torticollis | POD1 | 1 day | Traction | Full recovery |
| Deichmueller et al. 2010 [2] | 7, M | None | ND | Tympanoplasty | ND | Left | ND | | Painful torticollis | ND | ND | Antibiotics, analgasia | ND |
| Pilge et al. 2011 [7] | 11, F | None | SNHL | Cochlear implantation | ND | ND | ND | | Painful torticollis | Immediatery after surgery | 2 months | Manual repositioning, collar | Full recovery |
| Kim SY et al. 2011 [10] | 10, M | None | Chronic otitis media | Tympanoplasty | 90 | Left | Right | | Painful torticollis | POD1 | 4 days | Traction | Full recovery |
| Tauchi et al. 2013 [5] | 7, F | None | Cryptopia | Otoplasty | 180 | Right → left | Right | | Painful torticollis | POD3 | 1 day | Traction, manual manipulation collar | Full recovery |
| Romdoo et al. 2014 [6] | 15, M | Surgery for sagittal craniosynostosis | Tympanic membrane perforarion | Tympanoplasty | ND | Left | Right | | Painful torticollis | Immediatery after surgery | 6 months | Surgery | Persistent fusion |
| Nakashima et al. 2016 [9] | 7, M | None | SNHL | Cochlear implantation | 170 | Right | Left | | Painful torticollis | POD1 | 4 days | Traction, collar | Full recovery |
| Present case 1 | 7, M | None | Cholesteatoma | Tympanoplasty | 214 | Right | Left | | Painful torticollis | POD1 | A few hours | Traction | Full recovery |
| | | | Cholesteatoma | Tympanoplasty | 170 | Right | Left | 〇 | Painful torticollis | POD1 | A few hours | Traction | Full recovery |
| Present case 2 | 5, F | None | SNHL | Cochlear implantation | 226 | Left | Right | | Painful torticollis | POD1 | A few hours | Traction | Full recovery |
